# Alternative splicing is a driving force that tunes metabolic adaptations to virulence traits in the dermatophyte *Trichophyton rubrum*

**DOI:** 10.3389/fcimb.2025.1645525

**Published:** 2025-09-10

**Authors:** Marcos E. Ramos Lopes, João Neves-da-Rocha, Pablo R. Sanches, Vanderci M. Oliveira, Antonio Rossi, Nilce M. Martinez-Rossi

**Affiliations:** Department of Genetics, Ribeirão Preto Medical School, University of Sao Paulo, USP, Ribeirão Preto, SP, Brazil

**Keywords:** transcription factor, fungal pathogen, metabolism, intron retention, alternative splicing, PacC, Ap1, Con7

## Abstract

**Introduction:**

Alternative splicing (AS), a common process in pathogenic fungal species, is not fully understood. We hypothesized that AS is a critical regulatory mechanism that enables species to undergo continuous adaptations during interactions with challenging host environments.

**Methods:**

We utilized the model species *Trichophyton rubrum* to contextualize the role of AS in fungal physiology and virulence. We performed transcriptome-wide splicing analysis to search for AS events in RNA-sequencing data of *T. rubrum* grown in keratin. This scenario mimicked infection *in vitro* and allowed us to map biologically relevant splicing events.

**Results and discussion:**

Overall, the results showed that AS is recruited to regulate approximately 12.6% of the *T. rubrum* genome under an infection-like scenario. We extended this analysis to *ex vivo* infection models of *T. rubrum* grown on human nails and cocultured them with human HaCaT keratinocytes. We found that AS affects a wide range of cellular processes, including amino acid and carbohydrate metabolism, cell signaling, protein folding and transport, transcription, and translation. We showed that transcription factors such as PacC and Ap1 govern the major features of fungal virulence and metabolism and are controlled by the spliceosome machinery under different infection-like conditions.

**Conclusions:**

Our data indicate that mRNA isoforms originating from AS contribute to the adaptation of *T. rubrum*, demonstrating that AS of transcription factor genes plays a central role in fungal pathogenesis. The transcription and splicing machinery tune fungal physiology to achieve an optimal metabolic balance in virulence traits during infection.

## 1 Introduction

Dermatophytes are opportunistic pathogens that specifically colonize keratinized tissues. These pathogens engage in complex coevolutionary dynamics with their hosts, shaping their ability to colonize and infect keratinized tissues, such as the skin and nails, while influencing host immune responses (Deng et al., 2023; Gupta et al., 2023; Peres et al., 2010). Anthropophilic dermatophytes are an increasingly relevant medical concern affecting approximately 25% of the world’s population and cause chronic infections that can progress to invasive diseases in patients who are immunocompromised (Martinez-Rossi et al., 2021). *Trichophyton rubrum* is the most prevalent and clinically significant species worldwide, accounting for over 60% of all dermatophytosis cases (Zheng et al., 2020; Wang et al., 2021). Moreover, the emergence of resistant strains poses a challenge to the effective management of fungal infections (Bristow and Joshi, 2023; Martinez-Rossi et al., 2018; Kruithoff et al., 2023). Continuous adaptation of these organisms and their ability to resist conventional antifungal treatments emphasizes the need for ongoing research to determine the mechanisms underlying their infection persistence and develop novel therapeutic approaches.

Carbohydrate substrates are scarce in host tissues; therefore, dermatophytes degrade more complex molecules during infection. The main sources of available nitrogen and carbon are proteins, notably keratin. *T. rubrum* metabolic plasticity occurs via nitrogen catabolite repression, which is coordinated by the GATA zinc-finger transcription factors (TFs) (Martins et al., 2020). This process allows the use of complex nitrogen sources when preferential nitrogen sources such as glutamine are unavailable. However, keratin has a rigid structure formed by disulfide bonds, which prevent the usual proteolytic digestion of this molecule. Dermatophytes can produce urea and sulfites secreted by the Ssu1 efflux pump, which reduces covalent bonding (Martins et al., 2020; Grumbt et al., 2013). Keratin degradation and the metabolism of specific amino acids such as glycine to generate acetyl-CoA promote ammonia release and consequent alkalinization of the medium, which can increase pH values to as high as 9.0 (Ferreira-Nozawa et al., 2003; Maranhão et al., 2007). As the infection progresses, repression/activation of pH-regulated pathways and enzymes occurs in response to cell demands and environmental changes.

In this scenario, effective adaptation during infection requires fungi to coordinate diverse metabolic responses. Fungal cells activate transcriptional programs according to the surrounding conditions, such as cellular stress and host infection sites (Martinez-Rossi et al., 2021; Park et al., 2013). Arguably, the most important components governing these response programs are TFs, which regulate gene expression through hierarchical interactions between signaling and response networks (Song et al., 2016). In dermatophytes, many attributes related to virulence and susceptibility to stressors, as well as tolerance to different antifungals, are regulated by TFs such as PacC, Dnr1, Ap1, HacA, SteA, and StuA (Kröber et al., 2017; Yamada et al., 2006; Ferreira-Nozawa et al., 2006; Peres et al., 2022; Bitencourt et al., 2020; Lang et al., 2020; Martins-Santana et al., 2025).

The post-transcriptional regulation of gene expression by alternative splicing (AS) is a common process in fungi (Grützmann et al., 2014). AS may influence the activity of many genes in response to the various conditions imposed on these organisms (Grützmann et al., 2014; Mendes et al., 2018; Neves-da-Rocha et al., 2019; Lopes et al., 2022). Intron retention (IR) events are the most common type of AS in fungi (Grützmann et al., 2014; Kempken, 2013; Jeon et al., 2022; Li et al., 2021). In addition, important correlations exist between AS and distinct fungal species, with multicellular complexity, pathogen lifestyle, and a younger evolutionary age being associated with higher AS rates (Grützmann et al., 2014). Nevertheless, the extent of AS and the biological relevance of this post-transcriptional mechanism in fungal pathogenesis remain unclear. To investigate and correlate AS with fungal biology, we assessed global AS patterns in the transcriptome of *T. rubrum* exposed to keratin, which enabled mapping of biologically relevant AS events in an infection-like manner. We further performed functional enrichment analysis to investigate the physiological consequences of AS regulation. IR events in major fungal TFs were validated using *in vitro* and *ex-vivo* infection models to demonstrate the practical relevance of our findings. Because dermatophytes are representative filamentous pathogenic fungi in which AS is prevalent, this study improved the understanding of the strategies employed by pathogens to adapt to and colonize host tissues. Furthermore, our data provide critical fundamental information for guiding future studies on AS and medical mycology.

## 2 Materials and methods

### 2.1 *In vitro* culture conditions

*Trichophyton rubrum* strain CBS118892 from the Westerdijk Fungal Biodiversity Institute, Netherlands, was pre-cultivated in malt extract agar medium (2% glucose, 2% malt extract, 0.1% peptone, and 2% agar, pH 5.7) at 28°C for 21 days. Subsequently, 1 × 10^6^ conidia were germinated in 100 mL Sabouraud dextrose broth for 96 h and then transferred to Cove’s minimal medium at pH 5.0, containing either 50 mM glucose (Sigma–Aldrich, St. Louis, MO, USA) (control) or 0.5% (m/v) of powdered ox hull keratin as the carbon source and cultivated for 24 and 96 h (Peres et al., 2016). The cultures were filtered after each growth period, and mycelia were stored at −80°C until RNA extraction. All experiments were conducted in triplicate.

### 2.2 *Ex vivo* infection of human nail fragments

Human nail fragments obtained from healthy donors were autoclaved and placed in Eppendorf tubes. Conidia (1 × 10^4^ in 20 µL of sterilized water) of *T. rubrum* were mixed with the nail fragments (approximately 1 mm^2^). After 1 h of incubation at room temperature, 200 μL of sterilized water was added, and the tubes were incubated at 28°C for 96 h (Peres et al., 2016). The infected nail fragments were vortexed to release the fungal mycelia and extract total RNA. The supernatant containing fungal cells was centrifuged at 12,000 ×*g* for 10 min, frozen in liquid nitrogen, and stored at −80°C until RNA extraction. Fungi grown in glucose (described in Section 2.1) were used as a reference. The assay was performed in triplicate.

### 2.3 Coculture of *T. rubrum* with human keratinocytes (HaCaT)

For coculture experiments, 1 × 10^7^ conidia/mL and 2 × 10^5^ HaCaT cells/mL were incubated for 24 h in RPMI medium (Sigma–Aldrich, St. Louis, MO, USA) supplemented with 5% fetal bovine serum at 37°C in 5% CO_2_, as previously reported (Komoto et al., 2015). Plates containing fungal cells without keratinocytes were used as controls. The assay was performed in triplicate.

### 2.4 Extraction, cDNA synthesis, and RT-qPCR analysis

Total RNA was extracted using an Illustra Spin RNA Isolation Kit (GE Healthcare, Chicago, IL, USA) according to the manufacturer’s instructions. RNA samples were treated with DNAse I (Sigma-Aldrich, St. Louis, MO, USA) and converted to cDNA using a high-capacity reverse transcription kit (Applied Biosystems, Foster City, CA, USA). To evaluate the quality of the cDNAs, PCR was conducted using oligonucleotides flanking intron-1 of the constitutive gene encoding β-tubulin, and the results were visualized on an agarose gel.

Transcripts were quantified using a QuantStudio v. 3 Real-Time PCR System (Applied Biosystems, Foster City, CA, USA). Primers were designed within intron regions to detect IR events. Exon regions were used as primer annealing sites to evaluate total gene expression. The construction and quality analysis of the primers used in RT-qPCR assays were performed using the PrimerQuest Tool (http://www.idtdna.com/primerquest/Home/, accessed on June 12, 2023) and OligoAnalyzer Tool (https://www.idtdna.com/calc/analyzer, accessed on June 14, 2023) from Integrated DNA Technologies (IDT), and the BLAST tool (http://blast.ncbi.nlm.nih.gov/Blast.cgi, accessed on June 14, 2023).

The primers used and their standardized concentrations are listed in **Supplementary Table S1**. Reactions were performed using Power SYBR Green PCR Master Mix (Life Technologies, Carlsbad, CA, USA). Relative quantification was performed using the 2^−ΔΔCt^ method (Livak and Schmittgen, 2001), with *gapdh* and *rpb2* as reference genes for expression normalization (Jacob et al., 2012). The results are expressed as mean relative expression values from three independent replicates, with standard deviations. Alternatively, spliced transcripts were evaluated as a percentage of RNA isoforms, calculated using the ratio of mean ΔCT values of IR to total expression.

### 2.5 Global AS analysis and functional enrichment

High-throughput RNA-sequencing data were analyzed to assess transcriptome-wide AS regulation in *T. rubrum* exposed to keratin (Gene Expression Omnibus database, accession number GSE134406). Reads were mapped to the reference genome using STAR aligner v2.7.10a (Dobin et al., 2013). Reads mapped to multiple locations were excluded using the STAR – out Filter Multimap N max 1 parameter, and gene-level read counts were quantified using the STAR – quant Mode Gene Counts parameter. Visual inspection was performed using Integrative Genomics Viewer (IGV) v2.16.2 (Robinson et al., 2011).

The biological replicates were inspected using principal component analysis. We processed and analyzed the reads in R v4.3.1 using the ASpli Bioconductor package v2.10.0 to identify AS events (Mancini et al., 2021) and the DESeq2 Bioconductor package v1.40.2 to perform differential expression analysis (Love et al., 2014). The ASpli output file is shown in **Supplementary File S1**. Events with a Benjamini–Hochberg adjusted p-value lower than 0.05 and a log_2_ fold change greater than | ± 1.0| were considered significantly modulated. Functional categorization was conducted according to Gene Ontology (GO) terms assigned using the OmicsBox tools v3.1 (Conesa et al., 2005). The FunRich tool v3.1.4 (Pathan et al., 2015) was used to perform an enrichment analysis on the identified dataset.

### 2.6 *In silico* analyses

Data from Ensembl fungi were used to predict the transcripts and proteins resulting from conventional and alternative isoforms (https://fungi.ensembl.org/index.html, accessed on June 25, 2023). Reading frames and domains were predicted using the Expasy Translate tool (https://web.expasy.org/translate/, accessed on June 25, 2023) and InterPro database (https://www.ebi.ac.uk/interpro/ accessed on June 25, 2023), respectively. Graphical representations of the isoforms and resulting proteins were generated using Illustrator of Biological Sequence (IBS) v1.0 software (Liu et al., 2015).

To detect putative phosphorylation sites on serine, threonine, and tyrosine residues in the TFsproteic isoforms, the generated amino acid sequences were used as inputs to the NetPhos tool v3.1 (https://services.healthtech.dtu.dk/services/NetPhos-3.1/, accessed on June 27, 2023) (Chun et al., 2021). Conventional and alternative isoforms of the evaluated genes were used for the predictions, and sites with scores higher than 0.5 were defined as candidate phosphorylation sites.

## 3 Results

### 3.1 *Trichophyton rubrum* coordinates global AS patterns in the presence of keratin

We applied high-confidence statistical thresholds to evaluate AS in an infection-like scenario, which enabled mapping of bona fide AS events that could be critical for dermatophyte pathogenesis. AS analysis was primarily designed to identify IR events, which are the most prevalent type of AS in fungi (Grützmann et al., 2014). IR events influence translation and commonly change the open reading frame structure.

**Figure 1:**
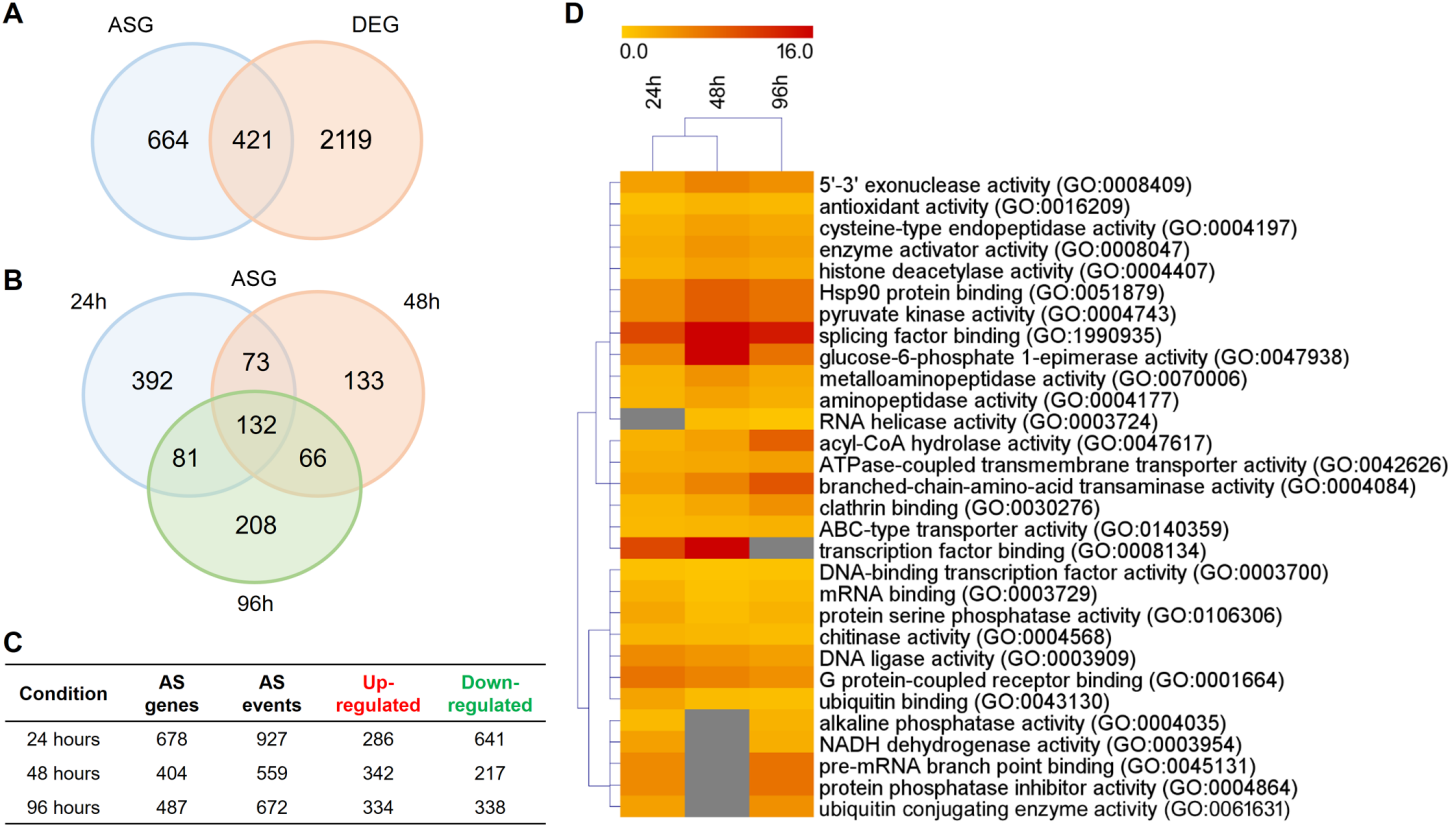
Distribution of genes modulated in response to keratin compared with the glucose (control). **(A)** Venn diagram of the number of alternative splicing genes (ASGs) and differentially expressed genes (DEGs) in response to keratin. **(B)** Venn diagram of alternative splicing (AS) events at 24, 48, and 96 h. **(C)** Total number of up- and down-regulated AS events at each time point. **(D)** Heatmap of functional enrichment analysis of ASGs based on Gene Ontology (GO) molecular function categories. The yellow-to-red color gradient indicates the fold-enrichment indices, which range from 0 to 16. Only the 30 most representative GO categories are displayed.

**Figure 2:**
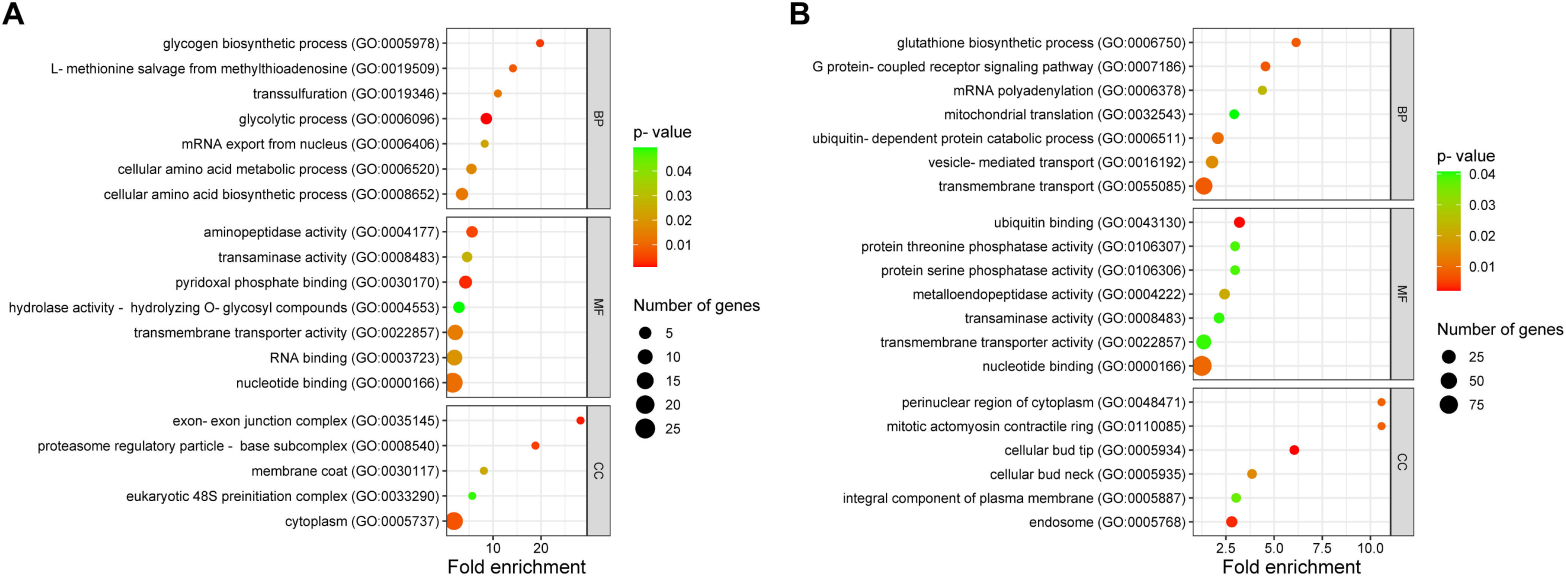
Functional characterization of genes with alternative splicing (AS) events in response to keratin. Functional annotation is categorized into biological processes (BP), molecular functions (MF), and cellular components (CC). **(A)** Functional characterization of 132 genes (from **Figure 1B**) that exhibited AS across all tested time points. **(B)** Functional categorization of genes with repressed AS in keratin. Sphere size represents the number of genes, whereas the color gradient, from red to green, indicates the p-value, ranging from smallest to largest.

To mimic infection progression and analyze AS over time, we compared fungal growth in keratin and glucose media at 24, 48, and 96 h. Overall, we detected AS events in 1085 genes, representing approximately 12.6% of the *T. rubrum* genome. The number of alternatively spliced genes (ASGs) was lower than that of differentially expressed genes (DEGs). Although most genes were exclusively regulated by AS or differential expression, 421 genes were simultaneously regulated by both processes (**Figure 1A**). The total number of ASGs represented a significant fraction of all gene expression changes in response to keratin, revealing that splicing plays a prominent role during *T. rubrum* infection (**Figure 1A**). Over time, AS events were prevalent at 24 h, and many ASGs were regulated at two or more time points (**Figures 1B, C**), reinforcing the importance of AS modulation in the infection process. Functional enrichment analysis of the genes that undergo modulation of AS expression in keratin revealed distinct molecular functions, such as splicing factor binding, helicase, kinase, phosphatase activities, and TF binding (**Figure 1D**). Remarkably, modulation of 132 ASGs at all incubation times indicated that these genes are involved in the virulence of *T. rubrum* and are mostly related to energy and amino acid metabolism (**Figures 1B**, **2A**).

**Figure 3:**
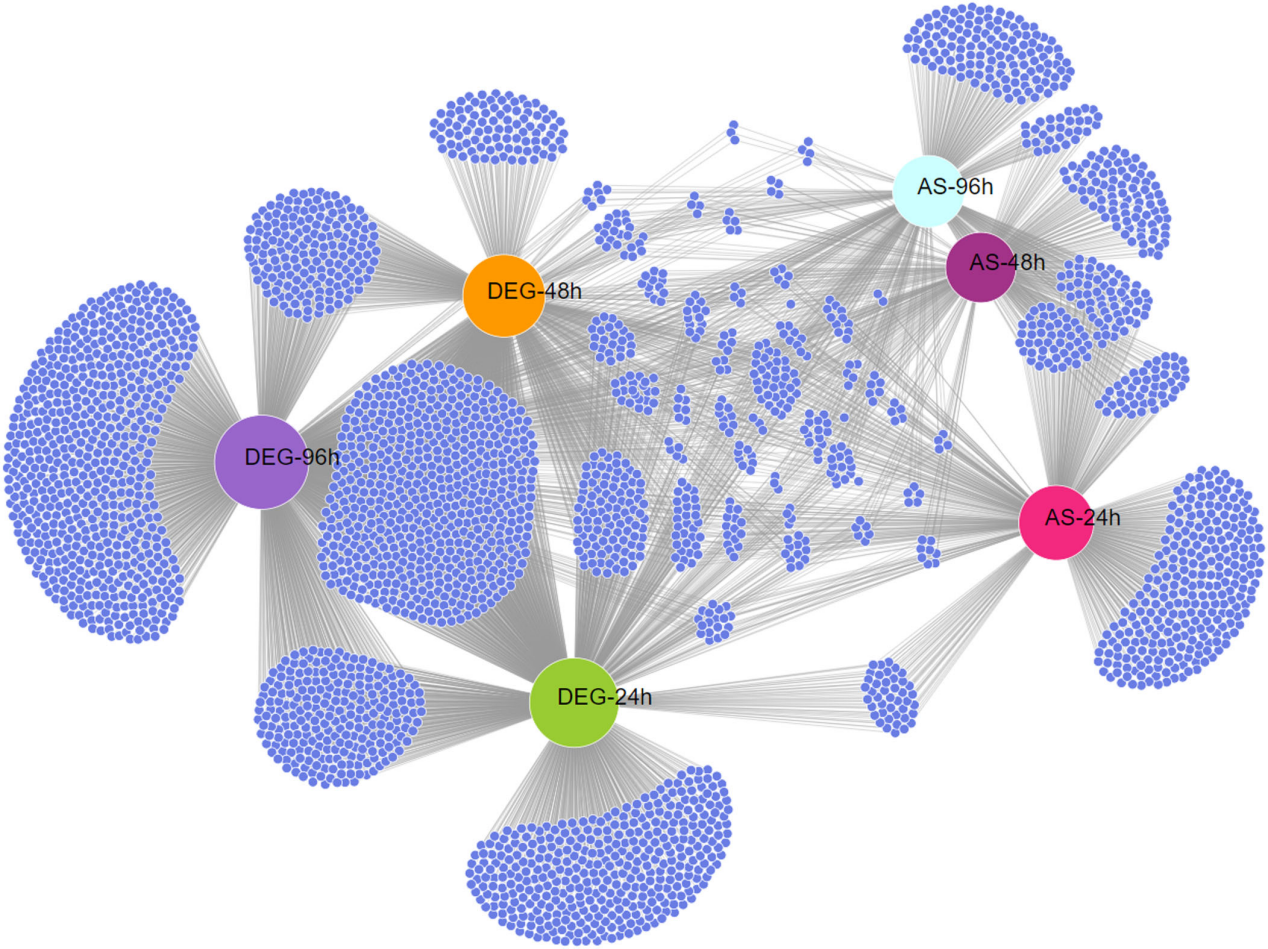
Expression network between differentially expressed genes (DEGs) and alternatively spliced genes (ASGs) in *Trichophyton rubrum* in response to keratin. Network representation of DEGs and ASGs across all experimental conditions. Small blue nodes represent individual genes, whereas gray lines indicate connections between regulatory mechanisms and conditions.

The results also revealed that a large number of AS events were repressed in response to keratin (**Figure 1C**), indicating that AS governs many processes related to cell signaling, transmembrane transport, energy metabolism, autophagy, and other processes important for fungal physiology under control and unstressed conditions (**Figure 2B**). Based on the gene expression network, despite regulatory overlaps, AS and DE are distinct processes that coordinate different aspects of fungal metabolism (**Figure 3**).

### 3.2 Keratin metabolism modulates expression and AS in TF genes

**Figure 4:**
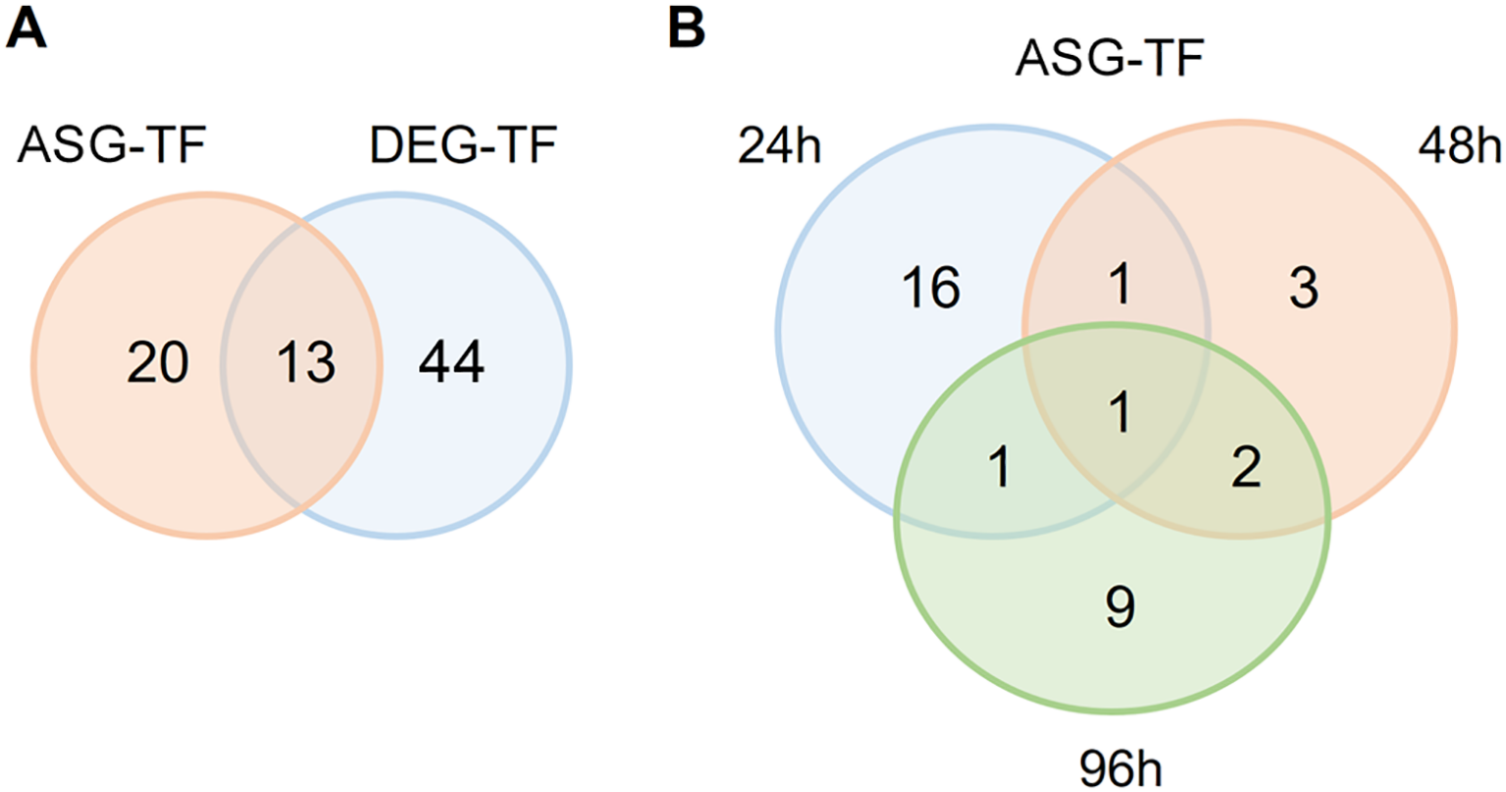
Venn diagrams showing the number of transcription factor (TF) genes differentially expressed (DEG) and alternatively spliced (ASG) in response to keratin. **(A)** Number of TF-coding genes showing alternative splicing (AS) events and were differentially expressed when *Trichophyton rubrum* was exposed to keratin. **(B)** Number of TF genes showing AS at different times of exposure to keratin.

RNA-sequencing data from *T. rubrum* exposed to keratin revealed the differential expression of 57 TF-coding genes (**Supplementary Figure S1**). Because TFs are key regulators of several cellular processes, we evaluated whether AS mechanisms, particularly IR, are involved in this regulation. Our analyses revealed that 33 TF genes presented IR in response to keratin, 13 of which also presented DE, and 20 were exclusively controlled by AS (**Figure 4**). These results demonstrate the importance of this regulatory layer in the functionality of this gene family.

**Figure 5:**
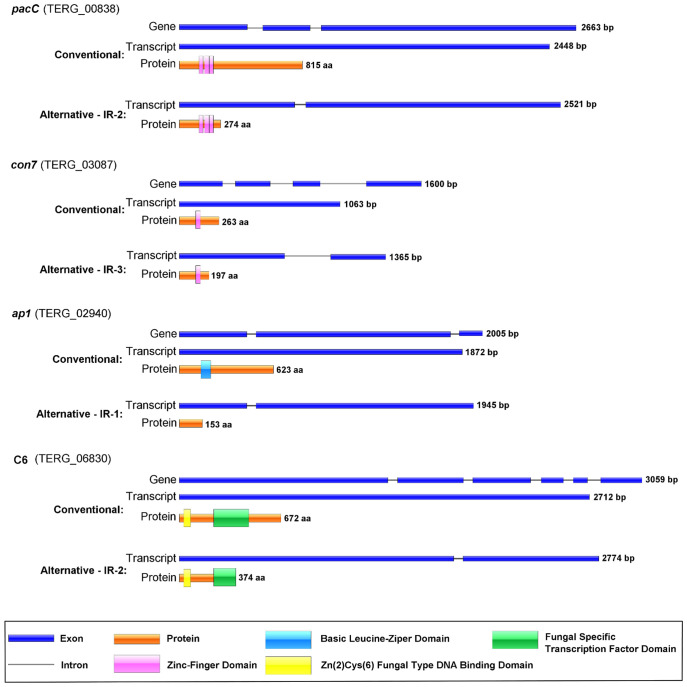
Schematization of transcription factor isoforms after conventional or alternative splicing. The transcripts and putative proteins resulting from each isoform are shown. Blue boxes represent exons, and introns are depicted as lines. Domains are indicated in the protein structure. IR shows the number of retained introns.

**Figure 6:**
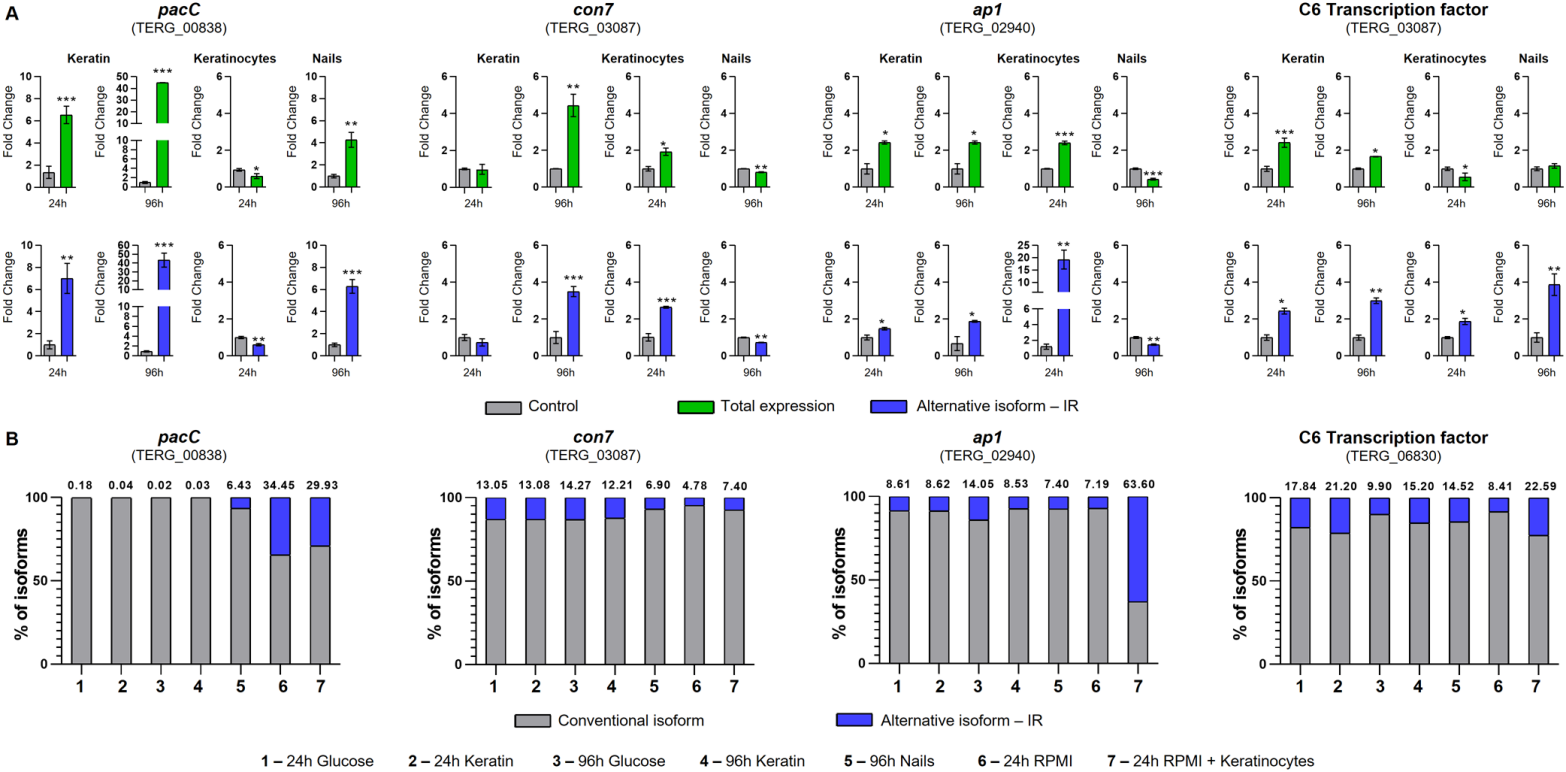
Expression of transcription factor isoforms of *Trichophyton rubrum* under *in vitro* and *ex vivo* conditions. **(A)** Expression analysis of *pacC*, *con7*, *ap1*, and *c6* isoforms in *T. rubrum* upon exposure to several challenges. The control conditions were as follows: keratin (glucose 24 and 96 h), keratinocytes (RPMI 24 h), and nails (glucose 96 h). Paired controls, indicated by gray bars in each graph, were used as modulation references. Statistical analyses were performed using *t*-tests. *p < 0.05, **p < 0.01, ***p < 0.001. **(B)** Intron retention (IR) percentage of *pacC*, *con7*, *ap1*, and *c6* transcripts of *T. rubrum* exposed to different conditions. The proportions of alternative isoform IR are represented in blue, and the conventional isoform is in gray. The numbers above each bar represent the percentage of alternate splicing isoforms.

We selected four TFs exhibiting IR and involved in virulence to determine the modulation of AS events under *ex vivo* conditions (keratinocytes and human nails) and compared them with those under keratin culture conditions. The four genes and their IR were *pacC* (TERG_00838, IR-2), *con7* (TERG_03087, IR-3), *ap1* (TERG_02940, IR-1), and *c6* (TERG_06830, IR-2). We employed *in silico* approaches to investigate the effects of IR on pre-RNA of the selected genes. IR events generated premature stop codons in the sequences of retained introns, resulting in specific outcomes such as changes in phosphorylation profiles and protein shortening with partial or total domain loss (**Figure 5**; **Supplementary Figure S2**). The presence and modulation of IR events were validated for all genes tested (**Figures 6A**). **Figure 6A** also shows that *pacC* was highly expressed in medium containing keratin or in human nail fragments, *con7* and *ap1* were overexpressed in keratin and keratinocytes, and *c6* was overexpressed only in keratin, indicating the specificity of each gene in the infection process.

To compare the levels of transcripts that exhibited IR with those that were fully processed (**Figure 6A**; **Supplementary Figure S3**), we calculated the percentage of IR under each experimental condition (**Figure 6B**). Under several assayed conditions (control or treatment), the *pacC*, *con7*, *ap1*, and *c6* transcripts presented IR levels greater than 5%, indicating that these ASs are not random events.

### 3.3 Splicing factor genes are induced upon exposure to keratin

**Figure 7:**
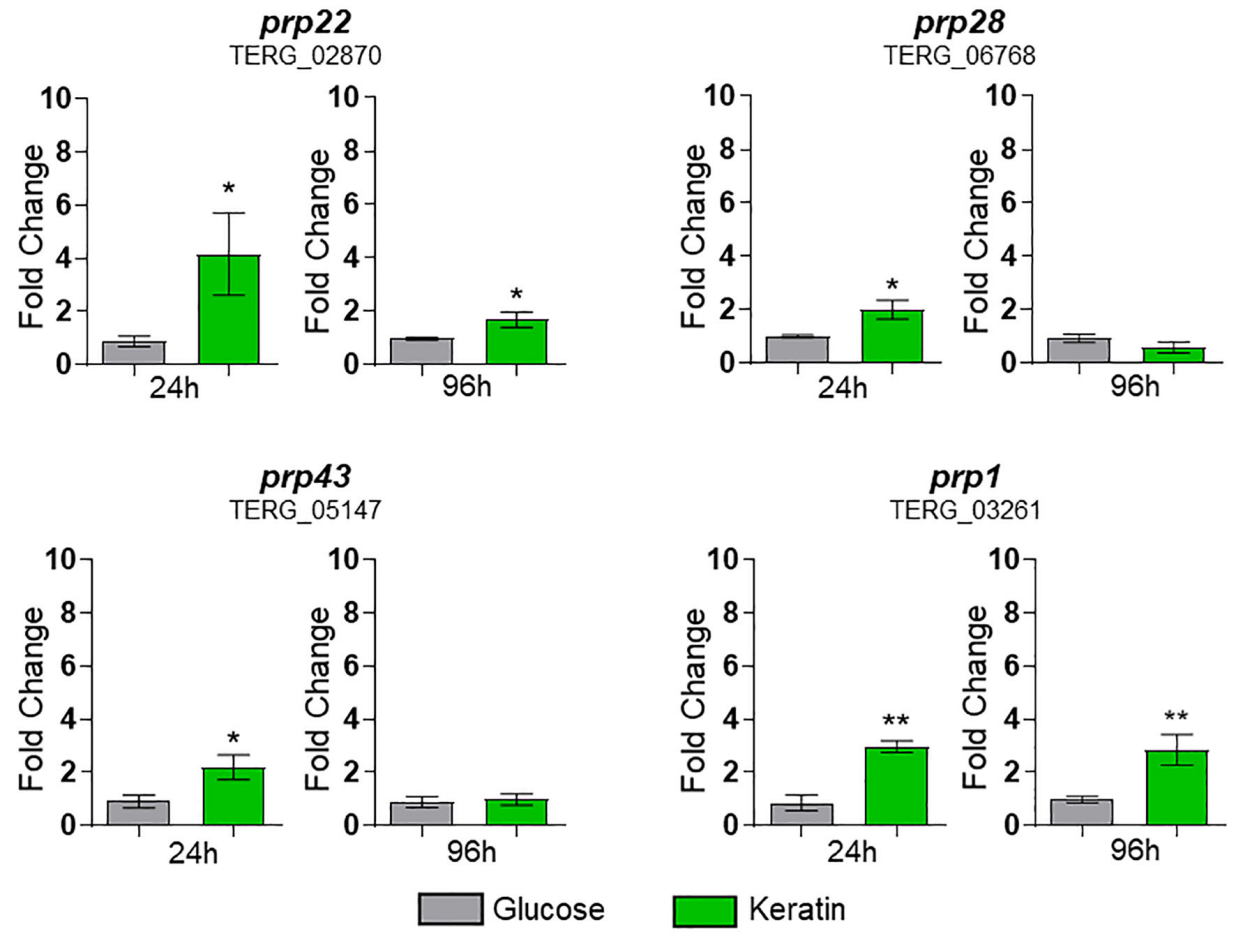
Expression of *prp*s (splicing factors) of *Trichophyton rubrum* exposed to keratin. Statistical analysis was performed using *t*-test. *p < 0.05, **p < 0.01. Paired controls, indicated by the gray bar on each graph, were used as a modulation reference.

To verify whether keratin metabolism modulates the expression of splicing factor genes, we assayed four genes following *T. rubrum* cultivation in keratin, using glucose as a control. These genes were induced by exposure to keratin, mainly at 24 h (**Figure 7**).

## 4 Discussion

We comprehensively evaluated the functional categories of genes that undergo AS in the human pathogen *T. rubrum* using a model that mimics infection. Our findings revealed that a large proportion of genes, approximately 12.6% of the genome, underwent AS, and approximately 39% of these genes are differentially expressed. This percentage of 12.6% is high considering the rate of AS observed in other pathogenic fungi, such as *Sclerotinia sclerotiorum* (5.6%) (Cheng et al., 2022), *Aspergillus oryzae* (8.55%) (Wang et al., 2010), and *Ustilago maydis* 3.6% (Ho et al., 2007), assayed under different conditions. Indeed, identification of TF genes that undergo many AS events in other pathogenic fungi suggests that AS regulates a broad spectrum of processes, highlighting the complex interplay of mechanisms regulating these genes in *T. rubrum*. Notably, at least 61% of ASGs did not exhibit differential expression, suggesting that AS plays a crucial role in regulating *T. rubrum* infection beyond the level of gene expression. These findings have significant implications for the understanding of pathogen–host interactions.

As a major step towards characterizing the role of the spliceosome machinery in dermatophytes, we assessed the global impacts of AS regulation on *T. rubrum* physiology. Functional enrichment analysis revealed that AS might contribute extensively to fungal metabolic adaptation during changes in nutrition sources, with direct impacts on the infection dynamics. During keratin degradation compared to glucose growth, AS influenced sensing and regulatory processes that are required for efficient physiological responses, such as kinase and phosphatase activities, transcription factors and splicing factors, transporters, enzyme activators, chaperones, and histone modifications (**Figure 1D**). Interestingly, considering the genes modulated in common in all time points in response to keratin, *T. rubrum* showed increased spliceosome influence over central carbon and nitrogen metabolism, affecting glycolysis, glycogen biosynthesis, amino acid metabolism, and proteasome function (**Figure 2A**). Those pathways indicate that dermatophyte physiology requires specific tuning of function to degrade complex substrates such as keratin, which is potentially influenced by nutrient limitation during keratin-based host colonization. Those findings are in accordance with previous data, which demonstrate keratin influence over dermatophyte central metabolism, including the control of nitrogen catabolite repression for regulating the TCA and urea cycles (Martins et al., 2020).

The number of ASGs in *T. rubrum* is particularly important during the initial phase of infection, when the pathogen must rapidly adapt to the new environment. Based on these findings, splicing factor genes such as *prp1*, *prp22*, *prp28*, and *prp43*, which are crucial in this process, were induced upon exposure to keratin, mainly at 24 h. This activation underscores the pivotal role of these four splicing factors in adaptation. Furthermore, AS events in TFs were present at all three tested time points, indicating that this phenomenon is an indispensable regulatory pathway for the adaptation, establishment, and maintenance of *T. rubrum* infection. The extensive range of categories that perform vital functions in cells under control of the splicing machinery in this infection-like context enhances the understanding of the crucial role of AS in fungal adaptation and pathogenicity. These findings may lead to the development of new strategies for combating fungal infections. The four selected TF genes revealed different expression profiles and IR in keratin medium, keratinocytes, and human nails. These results suggest that the fungus uses an intricate mechanism in its interaction with the host, depending on the tissue type and culture time.

**Figure 8:**
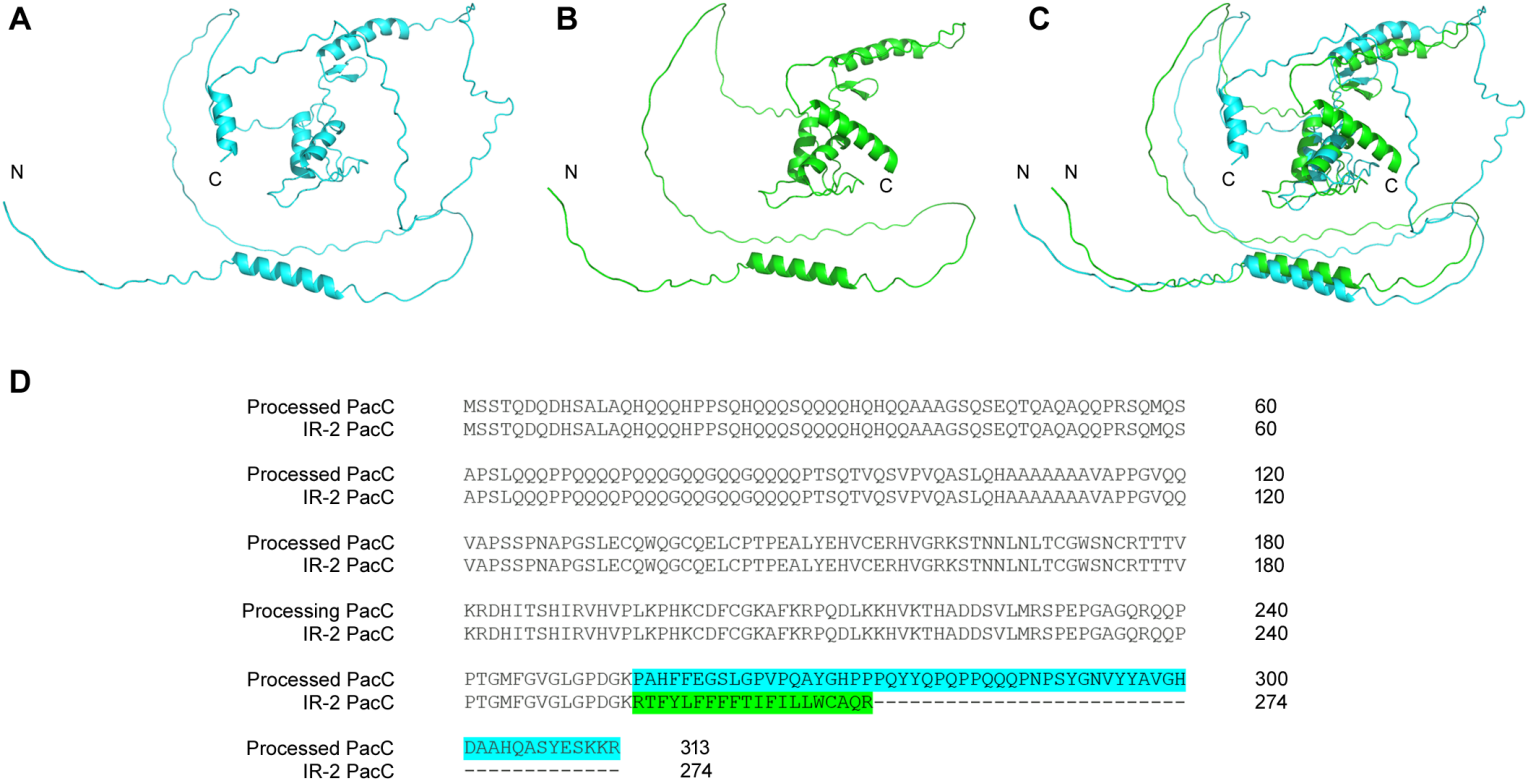
Comparison of putative PacC protein isoforms of *Trichophyton rubrum*. Three-dimensional structures of PacC protein enzymatically processed, in blue **(A)**, from IR-2, in green **(B)**, and their superposition **(C)**. Additionally, alignment of the amino acid sequences of these two protein variants is shown **(D)**.

The PacC signal transduction pathway, a crucial element in fungal infection, regulates the synthesis of a diverse range of enzymes and proteins with optimal activity at specific pH values dictated by fungal metabolism. This complex process enables the fungus to adhere to and penetrate host tissue, obtain nutrients, and subvert host defense mechanisms. Because human skin has an acidic pH, the first contact of dermatophytes during infection occurs in an acidic environment. We previously showed that the growth of *pacC* mutant strains of various fungi, including *T. rubrum*, is severely impaired under alkaline conditions (Ferreira-Nozawa et al., 2006; Trushina et al., 2013; Wiemann et al., 2009; Barda et al., 2020). The strong expression of *pacC* observed in keratin reinforces the involvement of this gene in keratin metabolism (Martins et al., 2020). The presence of IR-2 in *pacC* transcripts leads to the formation of protein isoforms that maintain three zinc-finger domains. In addition, *in silico* analyses indicated that the putative PacC protein generated by splicing (274 amino acids (aa)) resembled the activated form of the homolog protein of *Aspergillus nidulans* after proteolytic cleavage (254 aa) in terms of size, sequence, and domain number (three zinc finger domains) (**Supplementary Figure S4**). Functional activation of PacC in *A. nidulans* is a two-step proteolytic process, one of which is mediated by PalB, a calpain-family protease (Tilburn et al., 1995; Martinez-Rossi et al., 2012; Li et al., 2022), generating a minor protein containing three zinc fingers domains (Tilburn et al., 1995). Similarly, *in silico* data suggested that the putative version of PacC enzymatically activated in *T. rubrum* exhibited a conformation, size, and domain number similar to the putative isoform generated by IR-2 (**Figure 8**). Our RNA-seq data revealed high *pacC* expression, whereas differential expression of the *T. rubrum palB* homolog was not detected. This observation suggests the potential for alternative pathways to activate PacC protein at an acidic pH when culturing *T. rubrum* in glucose. Previous studies suggested that PacC protein in *A. nidulans* can be activated through an alternative pathway to PalB proteolysis due to the induction of an alternative palB isoform that is potentially nonfunctional, whereas PacC is functional (Trevisan et al., 2011). Our results suggest that IR-2 of the *pacC* pre-mRNA is an alternative mechanism to enzymatic activation of PacC at acidic pH in *T. rubrum*.

Although IR-3 in the *con7* transcripts resulted in a minor protein (197 aa) compared to the conventional isoform (263 aa), the zinc-finger domain was present in both isoforms. The prediction of phosphorylation sites in Con7 protein isoforms also revealed interesting differences between the two isoforms. These isoforms share 20 phosphorylation sites on the serine, threonine, and tyrosine residues. However, the alternative isoform had seven distinct phosphorylation sites resulting from the alternatively generated amino acid sequence, with five serine and two threonine residues (**Supplementary Figure S2**). These findings have important functional implications. Changes in the phosphorylation profile of TFs represent a mechanism employed by cells to mediate the activity of these proteins in response to environmental changes by regulating their transcriptional activity, cellular localization, stability, protein–protein interactions, DNA binding, and coregulators (Riedl and Egly, 2000; Whitmarsh and Davis, 2000; Zhou et al., 2020). Moreover, the IR-3 of con7 transcripts of *T. rubrum* was more prevalent when the fungus was cultured with keratinocytes than in the control (**Figure 6B**), suggesting a role of this isoform in tissue-specific interaction with the host. The role of this gene in fungal virulence and host interactions has been revealed in *Fusarium oxysporum* (Ruiz-Roldán et al., 2015). *con7* in *Magnaporthe grisea* and *F. oxysporum* also have splicing variants, including isoforms generated through IR during vegetative growth and infection (Odenbach et al., 2007; Ruiz-Roldán et al., 2015).

IR-1 in *ap1* transcripts generated a small protein of 153 amino acids lacking the essential leucine zipper domain, indicating loss of its function as a transcriptional regulator. However, notably, IR-1 isoform showed a high percentage among *ap1* transcripts when cultured with keratinocytes (63%), suggesting that this isoform is essential for interactions with HaCaT cells (**Figure 6B**). This alternative isoform may lead to the formation a putative mini-protein without a typical Ap1 domain. However, we cannot rule out the possibility that the RNA produced participates in fungal interactions with keratinocytes. As previously reported, the Δ*ap1* mutant of *T. rubrum* exhibited increased growth in cocultures with keratinocytes and nail fragments compared to the wild type, suggesting that *ap1* participates in the negative control of virulence-related attributes and contributes to the chronicity of infection caused by this dermatophyte (Peres et al., 2022). The regulation of fungal virulence traits affects the chronicity of the disease and evasion of the host immune system (Martinez-Rossi et al., 2021; Peres et al., 2022). We hypothesized that the correct balance in the production of these *ap1* isoforms contributes to the control of the chronic infection.

The presence of IR-2 in the *c6* isoforms resulted in a minor protein, with a partial loss of the fungal-specific TF domain. However, the Zn(2) Cys(6) DNA-binding domain was maintained, suggesting that the function of this putative protein differed from that of the conventional isoform. In keratin, human nails, and keratinocytes, the IR-2 isoform of *c6* was more prevalent than in its respective controls, suggesting that it functions under infectious conditions (**Figure 6B**). These data revealed the post-transcriptional regulation of the mechanisms involved in fungal pathogenicity. In *Magnaporthe oryzae*, TFs are a vital group of genes regulated by AS during fungal infections, with the C6 TF group being the most common (Jeon et al., 2022). The C6 TF of *Aspergillus fumigatus* (AFUB_043270) exhibits IR, which is a potential mechanism of self-protein activation control (Sieber et al., 2018). These data highlight the importance of C6-type TF AS events in several fungal infections.

Finally, the presence of alternative isoforms, even under control conditions (glucose or RPMI), indicates that infection conditions do not always dictate the presence of AS but instead influence the proportion of these events. These data underscore the importance of investigating the proportion of alternative isoforms compared to total isoforms, providing precise quantification of this phenomenon.

In conclusion, our data for these TF genes validate AS events and corroborate the idea that pathogenic fungi show different AS patterns in their infection environments (Jeon et al., 2022; Sieber et al., 2018; Cheng et al., 2022; Ibrahim et al., 2021). Our results also improve the understanding of the involvement of TF isoforms in this process, as *pacC*, *con7*, *ap1*, and *c6* are crucial TFs for virulence and fungal adaptation to the host environment (van der Does and Rep, 2017; Wang et al., 2024; John et al., 2021). Exposure of the fungus to keratinolytic substrates induces the modulation of transcriptional and post-transcriptional responses, particularly of virulence factors and metabolic regulation. The regulatory amplitude exerted by AS under infection-like conditions suggests the impact of this mechanism on providing adequate responses to *T. rubrum* pathogenesis. The consequences observed in the TF genes from this regulation clarify how this mechanism contributes to the strategies employed by the fungus to adapt and colonize the host. This study provides a foundation for future fungal infection management by improving the understanding of the molecular mechanisms employed by pathogens to infect their hosts.

## Data Availability

The data presented in the study are deposited in Gene Expression Omnibus (GEO) database under accession number GSE134406.
