# Supplementary material for: Alternative splicing is a driving force that tunes metabolic adaptations to virulence traits in the dermatophyte Trichophyton rubrum
Source: Front Cell Infect Microbiol. 2025 Sep 10;15:1645525. doi: 10.3389/fcimb.2025.1645525 (PMC12457390; doi:10.3389/fcimb.2025.1645525)
Supplement: Supplementary file 2 [file DataSheet1.pdf]

Supplementary Material

Supplementary Figures and Table

Supplementary Table 1 List of primers of RT-qPCR used in this work.

Gene/isoform	Sequence	Concentration	Reference
rpb2	F: TGCAGGAGGTTTGATGAAGA<br>R: GCTGGGAGGTACTGTTTGATCAA	300 nM	Jacob et al, 2012
gapdh	F: GCGTGACCCAGCGATGTAGT<br>R: CGGTGGACTCGACGATGTAGT	200 nM	Jacob et al, 2012
pacC<br>Intron-2 retention	F: TTCTATTGTGGTGTGCTCA<br>R: TAATACTGTGGTGGAGGGT	100 nM	This work
pacC<br>Total expression.	F: ACTCCAGAGACTACGTGACTATG<br>R: AGTCTTCCTGCTTGTCTTCG	200 nM	This work
con7<br>Intron-3 retention	F: TGCTGTCTCTCTCCTATCTAC<br>R: CTGCACACTCCACCAAGAT	300 nM	This work
con7<br>Total expression.	F: CTCCTGGTGGTTTGGTCTAT<br>R: GCCCTGACCATAAGGAGAATG	100 nM	This work
ap1<br>Intron-1 retention	F: CGTAAGTGTCTTCTGAGCCTT<br>R: GAGCTTTCCGTTTCTATGACC	300 nM	This work
ap1<br>Total expression.	F: CCTCCCTAAGGACGATCA<br>R: CCAGAACAAATCAGGTGGA	100 nM	This work
TERG_06830 *c6*<br>Intron-2 retention	F: CCCAGACACAGTCTCATCATTA<br>R: GTGGGAAGAGGAACGTCATAC	100 nM	This work
TERG_06830 *c6*<br>Total expression	F: CTGAGATTCTTGGCGATCTTCT<br>R: GTCTTCCCACCGATCCAAAT	300 nM	This work
TERG_02870<br>*prp22*	F: AAAGTCATCATCGTCTCTAACCAAC<br>R: GGATCATAGGCTCGCAGTTT	100 nM	This work
TERG_05157<br>*prp43*	F: CGTACTCGACCTGGGAAATG<br>R: ATCCAGAACCGTGGAAGATAAG	200 nM	This work
TERG_06768<br>*prp28*	F: GCCCAGCAGATCGAAATA<br>R: GCTCCGTTGCGTAGATTA	200 nM	This work
TERG_03261<br>*prp1*	F: AGAGAACATCCGCCTCAA<br>R: GCTTCGATCCAGAGTCTAGT	100 nM	This work

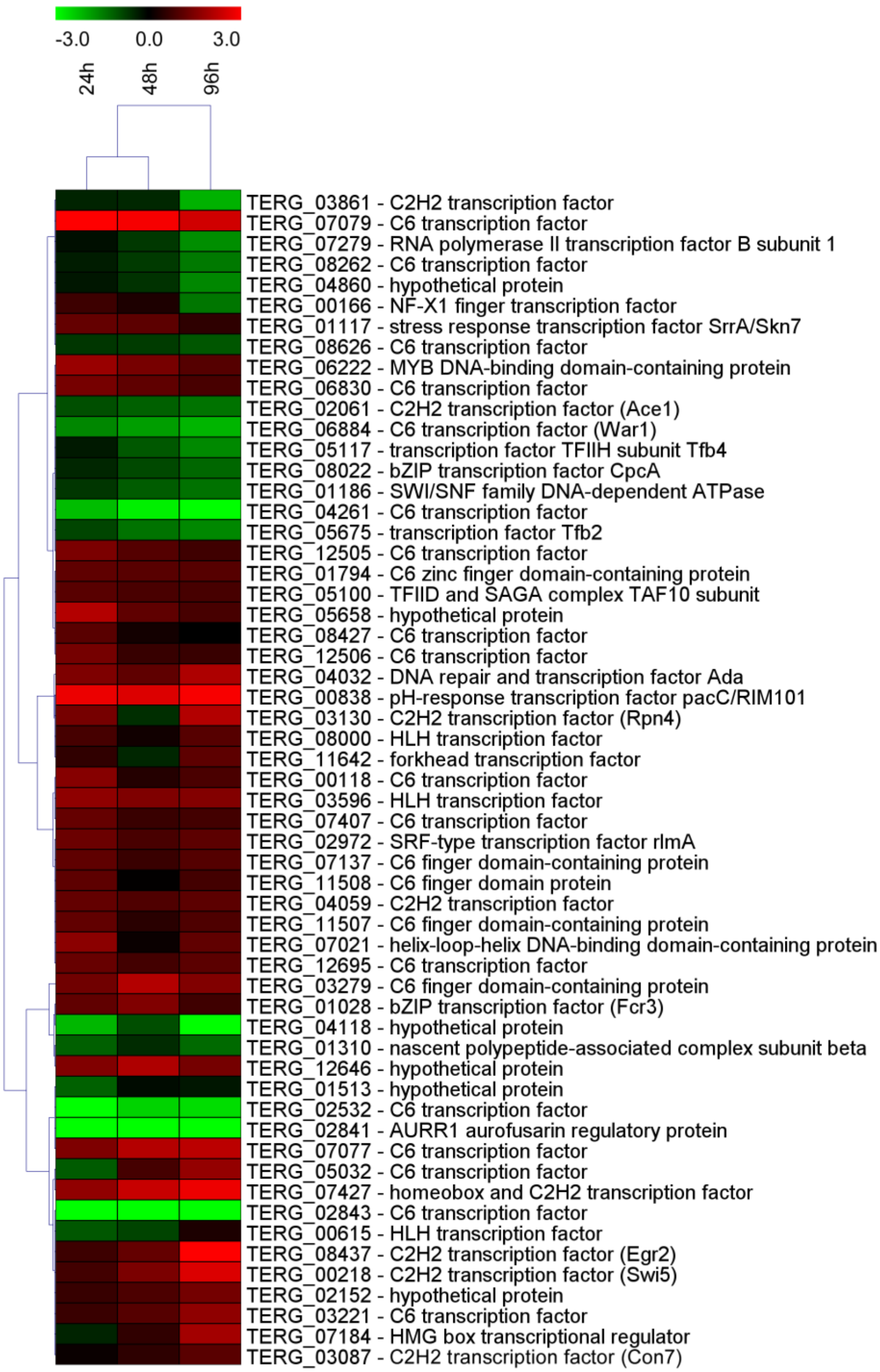

Supplementary Figure 1

Heatmap and hierarchical clustering of gene expression data. Gene encoding transcription factors of *Trichophyton rubrum* that are differentially expressed in response to keratin (24, 48, and 96 h of culture). The color scale from green to red indicates the lowest to the highest gene expression.

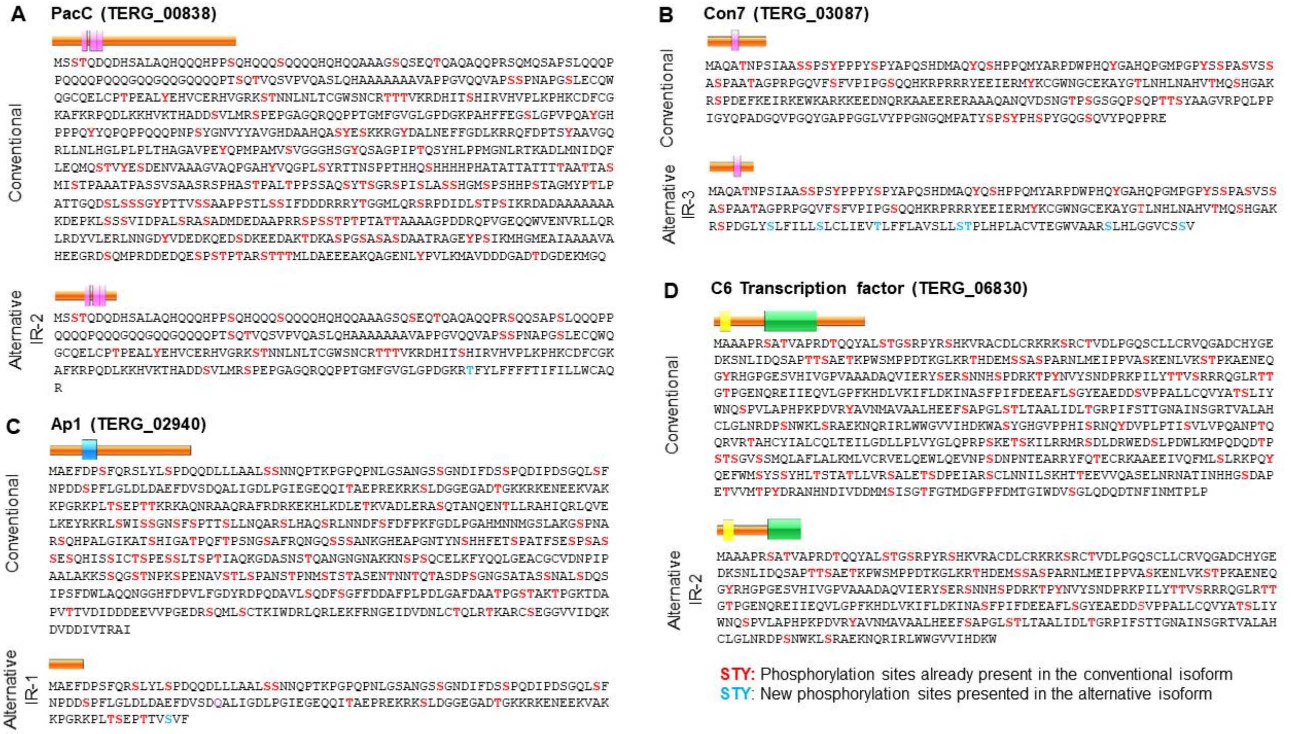

Supplementary Figure 2

Prediction of phosphorylation sites in protein isoforms of transcription factors in *Trichophyton rubrum*. The amino acid sequences of different transcription factor isoforms are presented. The amino acids highlighted in color (red and blue) are putative phosphorylation residues on serine (S), threonine (T), and tyrosine (Y). The residues in red are those already found in conventional isoforms, whereas those in blue are new residues presented in alternative isoforms.

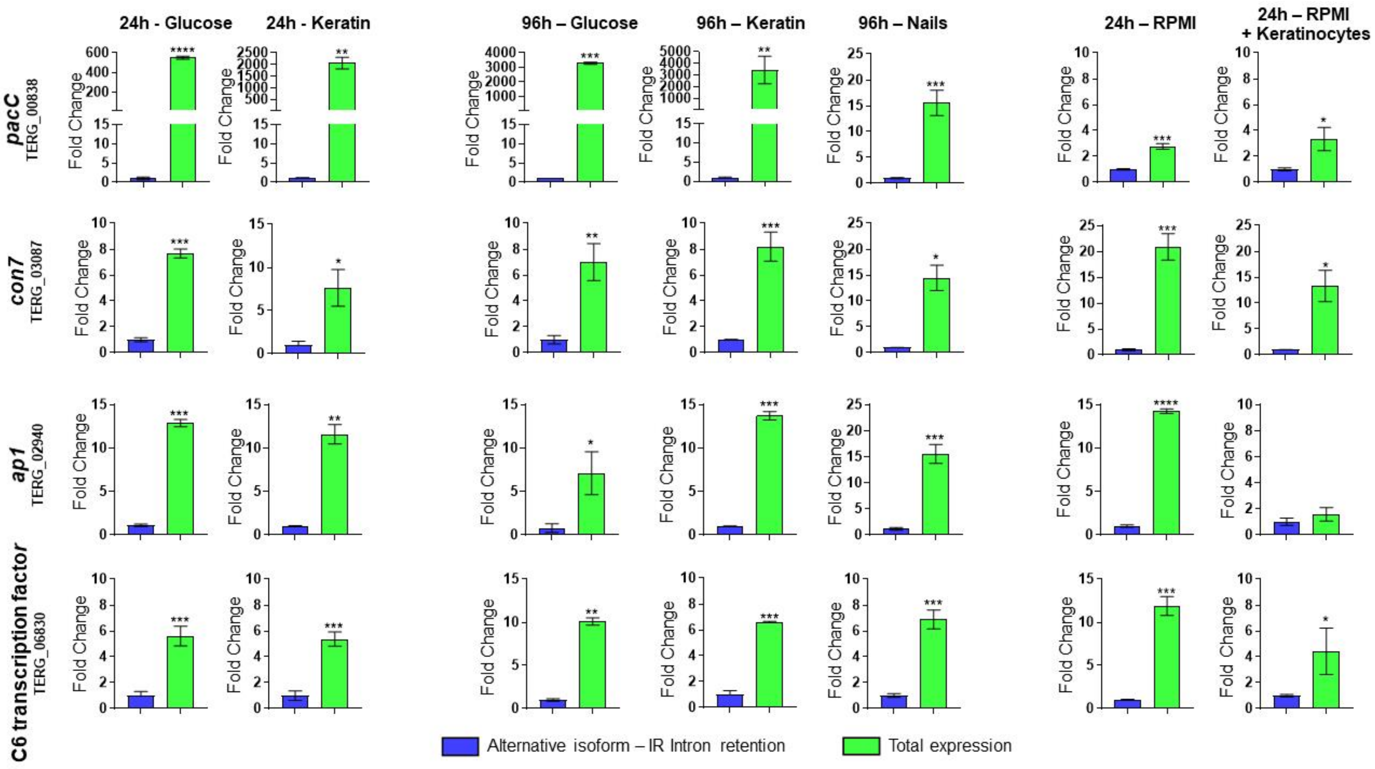

Supplementary Figure 3

Direct comparison between total gene expression and intron-retaining (IR) isoforms of transcription factor genes in *Trichophyton rubrum* under distinct experimental conditions. Total gene expression (green bars) was compared to IR isoforms (blue bars) for *pacC*, *con7*, *ap1*, and *c6* transcripts. IR was used as the paired reference for each condition. Statistical analysis was performed using *t*-test. $*p < 0.05$, $**p < 0.01$, $***p < 0.001$.

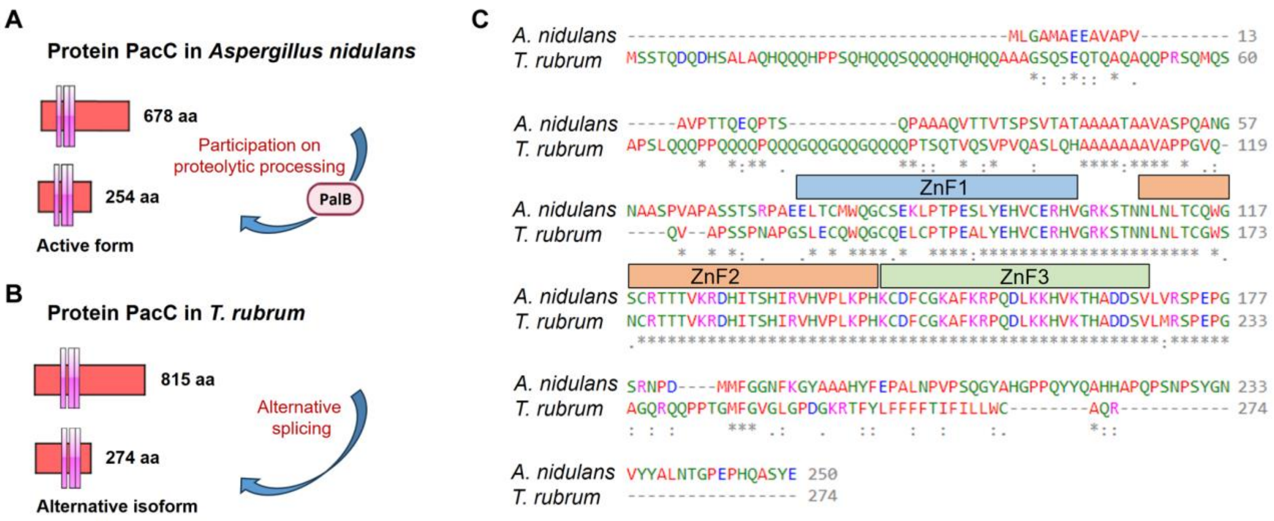

Supplementary Figure 4

Schematization and alignment of PacC protein resulting from proteolytic processing and alternative splicing (AS) in *Aspergillus nidulans* and *Trichophyton rubrum,* respectively. **(A)** Activation of PacC protein in *A. nidulans* following proteolytic processing is mediated by PalB, resulting in a minor protein with three zinc-finger domains. **(B)** Putative activation of PacC protein resulting from AS (IR-2 in *T. rubrum*). The two processes generate similar proteins in terms of size, conservation, and number of DNA-binding domains (zinc finger domain). **(C)** Alignment of PacC proteins originated from these two molecular processes. The blue, orange, and green rectangles represent the location of zinc fingers 1, 2, and 3 DNA-binding domains, respectively.
